# How to apply clinical cases and medical literature in the framework of a modified “failure mode and effects analysis” as a clinical reasoning tool – an illustration using the human biliary system

**DOI:** 10.1186/s13256-016-0850-6

**Published:** 2016-04-06

**Authors:** Kam Cheong Wong

**Affiliations:** 1 Bathurst Rural Clinical School, Western Sydney University, Bathurst, NSW Australia; 2 School of Rural Health, University of Sydney, Orange, NSW Australia; 3 George Street Medical Practice, Bathurst, NSW Australia

## Abstract

**Background:**

Clinicians use various clinical reasoning tools such as Ishikawa diagram to enhance their clinical experience and reasoning skills. Failure mode and effects analysis, which is an engineering methodology in origin, can be modified and applied to provide inputs into an Ishikawa diagram.

**Method:**

The human biliary system is used to illustrate a modified failure mode and effects analysis. The anatomical and physiological processes of the biliary system are reviewed. Failure is defined as an abnormality caused by infective, inflammatory, obstructive, malignancy, autoimmune and other pathological processes. The potential failures, their effect(s), main clinical features, and investigation that can help a clinician to diagnose at each anatomical part and physiological process are reviewed and documented in a modified failure mode and effects analysis table. Relevant medical and surgical cases are retrieved from the medical literature and weaved into the table.

**Results:**

A total of 80 clinical cases which are relevant to the modified failure mode and effects analysis for the human biliary system have been reviewed and weaved into a designated table. The table is the backbone and framework for further expansion. Reviewing and updating the table is an iterative and continual process. The relevant clinical features in the modified failure mode and effects analysis are then extracted and included in the relevant Ishikawa diagram.

**Conclusions:**

This article illustrates an application of engineering methodology in medicine, and it sows the seeds of potential cross-pollination between engineering and medicine. Establishing a modified failure mode and effects analysis can be a teamwork project or self-directed learning process, or a mix of both. Modified failure mode and effects analysis can be deployed to obtain inputs for an Ishikawa diagram which in turn can be used to enhance clinical experiences and clinical reasoning skills for clinicians, medical educators, and students.

Clinicians, medical educators, and medical students use various clinical reasoning tools such as Ishikawa diagram (which is also known as “cause-and-effect diagram”) to enhance their clinical experience and reasoning skills. The methodology of applying an Ishikawa diagram in a clinical setting is illustrated in another article [1]. The methods that can be applied to gather information for an Ishikawa diagram include brain storming, focus group discussion, interview, survey, and literature searches and review are discussed in a book chapter [2]. This article illustrates how to modify and apply failure mode and effects analysis (FMEA) to provide inputs into an Ishikawa diagram which in turn can be used as a clinical reasoning tool.

FMEA is a tool developed by engineers to systematically assess a complex design or process in order to identify elements that have a risk of failure [3]. In the late 1940s, FMEA was established and deployed by reliability engineers to identify potential failures in military systems [4]. Simplistically, the FMEA approach includes a meticulous study on the mode or mechanism by which a failure may occur and the effect(s) that it may cause. The severity of the effect (S), the probability of a failure occurring (O), and the probability that the failure would not be detected (D), are computed or estimated. Then, a risk priority number (RPN) is calculated by multiplying S, O, and D. The RPN is then used to prioritize the remedial and/or preventive measures. The FMEA approach is an ongoing iterative process. It should be updated when there is a change in the process or design, or when there is a failure or when a near-miss failure occurs. The ultimate objective of an FMEA is to provide a platform for the prevention, or at least reduce the likelihood and improve the detection, of failure in a system.

How can I relate this engineering approach to an application in medicine? In this article, failure is defined as an abnormality caused by infective, inflammatory, obstructive, malignancy, autoimmune and other pathological processes. I may not be able to relate the FMEA approach to the entirety of a complex human being with multidimensional complexities including psychosocial components, when an individual presents with a clinical manifestation (that is, a “failure”). Nonetheless, I am illustrating an application of the FMEA approach to a specific subsystem in a human. The “design or anatomy” of a human body and its underlying physiological processes have a potential risk of failing at its various parts anatomically and physiologically. The effect(s) of the failure could be manifested as clinical features (symptoms and signs). The severity, occurrence, and detection of the failure are complex and difficult to be estimated to compute a RPN. Hence, I am excluding RPN in this FMEA approach which I call a “modified FMEA” (in short, mFMEA). The ultimate objective of mFMEA is to provide a methodology to clinicians, medical educators, and medical students, to integrate in their clinical reasoning process and to deploy relevant clinical cases to set the scenario for teaching and learning a specific topic.

The mFMEA approach is applicable for general practitioners (GPs)/family physicians, specialists in various fields (internal medicine, surgery, emergency, intensive care, and so on), medical educators, and medical students. The bottom line is that a patient will always present with symptoms and signs (clinical features or syndromes) that need to be analyzed and put in perspective in an individualized context. An experienced clinician such as a physician or specialist may reach a spot diagnosis or provisional diagnosis, and manage the patient accordingly in a reasonably efficient timeframe. In an experienced diagnostician, the clinical reasoning and diagnostic skill seem to have become second nature to him/her, and the skills may not be explicit to an observer. However, a junior clinician or medical student may start from the first principle to work out a list of differential diagnoses using various clinical reasoning tools such as brain storming, mind mapping, and the Ishikawa diagram/fishbone diagram or cause-and-effect diagram [1]. For example, if a patient presents with a main complaint of pain in the right upper quadrant (RUQ) of the abdomen, a clinician will have a list of differential diagnoses including cholecystitis, cholelithiasis, hepatitis, peptic ulcer, pancreatitis, and referred pain. These can be illustrated in an Ishikawa diagram (Fig. 1) which is subject to ongoing update. The Ishikawa diagram comprises “gastroenterology”, “other systems”, and “miscellaneous”. The biliary system is a branch under “gastroenterology”. The Ishikawa diagram will be continually expanded and refined based on other associated symptoms in the individualized context of the patient. Then, one may ask how a mFMEA approach would fit in a clinical setting. A mFMEA can provide inputs into an Ishikawa diagram. Once you have listed the common causes in an Ishikawa diagram via brainstorming, discussion, or self-directed learning process, you turn to mFMEA to explore the potential causes of the human biliary system (Fig. 1). I would like to elaborate this in the following paragraphs.

**Fig. 1:**
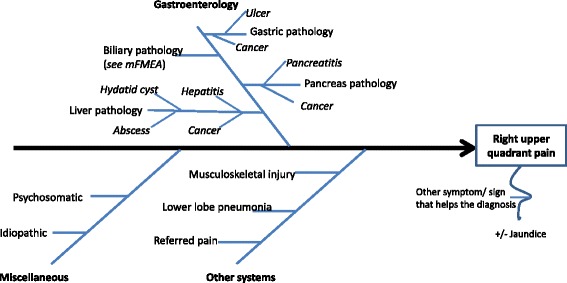
An Ishikawa diagram for a main presenting complaint of “abdominal pain in the right upper quadrant”. *mFMEA* modified failure mode and effects analysis

An English scientist, Richard Dawkins, once said, “Biology is the study of complicated things that have the appearance of having been designed with a purpose.” The notable quote underpins many biological and physiological processes within a human body; for example, the human biliary system. When we review the anatomical and physiological processes of the biliary system, we should ask “what is the potential failure and its effect(s)”, “the associated main clinical features”, and “investigations that can help a clinician to diagnose” at each anatomical part and physiological process, and document them in a mFMEA table (Table 1). The mFMEA table comprises six columns: “Anatomy and physiology”, “Potential failure or pathophysiological process”, “Effects of the failure”, “Main clinical features (symptoms and signs)”, “Investigation”, and “Note”. We can start with common knowledge found in the medical literature, and then proceed to search and extract the relevant medical and surgical cases (generally known as clinical cases in this article) to fill the mFMEA table. Establishing a mFMEA can be a teamwork project or a self-directed learning process, or a mix of teamwork followed by self-directed learning to continually update it.

**Table 1:** A modified failure mode and effects analysis of the human biliary system

Anatomy and physiology	Potential failure/pathophysiological process	Effects of the failure	Main clinical features (symptoms and signs)	Investigation^a^	Note
Gallbladder	Cholecystolithiasis (stones in the gallbladder) [6]	Non-obstructive [6]	Asymptomatic [6]	Abdominal X-ray and CT [6]	
	Cholecystitis –usually associated with obstruction of the outlet of the gallbladder by a gallstone [6]	The obstruction results in inflammation of the gallbladder, and may be complicated by secondary bacterial infection [6]	Murphy’s sign [6]	Abdominal ultrasound [6]	
	Cholecystitis associated with gallbladder torsion (unusual but critical) [7]	Torsion of the gallbladder around the cystic duct caused necrotic gallbladder [7]	Acute onset abdominal pain in the RUQ, with nausea and malaise [7]	Abdominal ultrasound and CT [7]	
	Malignancy – e.g. adenocarcinoma [8]	The gallbladder tumor invaded adjacent structures, caused acute and chronic cholecystitis with cholelithiasis and choledochoduodenal fistula [8]	1-month history of episodic nausea and vomiting, and epigastric pain on admission [8]	Abdominal ultrasound, CT, barium study [8]	
	Perforation – acute on chronic gallbladder inflammation possibly due to ischemia and bile stasis secondary to preoperative fasting [9]. Perforation – other causes can be Epstein–Barr virus [10], liver abscess [11], blunt abdominal trauma [12], and spontaneous [13]	Bile leaked into the peritoneum [9]	Umbilical pain and a bluish discoloration of the skin around a known umbilical hernia presumably due to tracking of bile within the abdomen [9]	Liver function test, laparotomy [9]	The patient had spontaneous acalculous gallbladder perforation [9]. This condition is rare but critical
	Gallbladder herniation: parastomal [14–18], incisional [19–21], spontaneous ventral [22, 23], epigastric [24], transdiaphragmatic herniation [25]	A midline abdominal hernia with small bowel loops, and a parastomal hernia containing the gallbladder [14]	Abdominal pain [14]	CT scan with oral Gastrograffin (sodium diatrizoate and meglumine diatrizoate) contrast [14]	
	Gallbladder torsion [17]	Torsion of the neck of the gallbladder with secondary suppuration leading to gangrenous changes in the gallbladder [17]	Abdominal pain [17]	Abdominal CT [17]	Other case of gallbladder torsion [26], a new case of gallbladder torsion within an incisional hernia [27], complete gallbladder torsion [28], gallbladder torsion caused acute cholecystitis [7]
	A gallstone enters via the Vater papilla and later increases in size [29]	Transition of a gallstone in the gastrointestinal tract leading to mechanical bowel obstruction (gallstone ileus) [29]	Nausea, vomiting and abdominal pain [29]	Abdominal CT, MRCP [29]	Other case: gallbladder-colon fistula [30] and cholecystogastric fistula [31]
	Biliary-enteric fistula e.g. gallbladder-duodenal fistula [8]	“Spontaneous biliary fistulas have been associated with gallbladder cancer; if they are the cause of cancer, or acomplication of it, this has not yet been defined.” [8]	1-month history of episodic nausea and vomiting, and epigastric pain on admission [8]	Abdominal ultrasound, CT, barium study [8]	
	Failure of the cystic bud to develop *in utero* [32]	Gallbladder agenesis [32]	Reduced appetite, unintentional weight loss, and a history of fatty food intolerance [32]	Abdominal ultrasound, CT, ERCP; preoperative MRCP should be considered in cases in which ultrasound suggests non-visualization of the gallbladder [32]	Agenesis of the gallbladder could be made only at laparotomy after having searched for, and excluded, an ectopic gallbladder [32]
Cystic duct	Obstruction such as due to gallstone; chronic obstruction due to other causes [33]	Hydropic gallbladder – accumulation of mucus in the distended gallbladder [33]	Abdominal pain in the RUQ [33]	Abdominal ultrasound and MRI [33]	
	Cystic duct obstruction (+/– cystic artery strangulation) due to torsion of the neck of the gallbladder [34]	Leading to mural ischemia/gallbladder becomes gangrenous, and may perforate [34]	Epigastric pain with involuntary guarding and rebound tenderness in the epigastrium and RUQ [34]	Abdominal ultrasound and CT [34]	Another case [35]
	Cystic duct infection e.g. streptococcal infection [36]	Streptococcal toxic shock syndrome; local necrosis in the cystic duct due to microcirculatory failure as a result of hypoperfusion and microthrombosis leading to perforation of the cystic duct [36]	Abdominal tenderness, rash and fever after an episode of pharyngitis* [36]	Liver function test, abdominal ultrasound and CT	* Refer to the case report for the time-course of the clinical features [36] Another case of cystic duct perforation with acalculous cholecystitis [37]
Common bile duct	Choledocholithiasis (stones in the common bile duct) [6]	Non-obstructive [6]	Asymptomatic [6]	ERCP [6]	
	Infection: bacterial [38]	Cholangitis [38]	Charcot’s triad (abdominal pain, fever and jaundice), nausea, and dark urine [38]	Ultrasound biliary tree, ERCP [38]	
	Infection: liver flukes from consumption of undercooked contaminated seafood [5]	The metacercariae then excyst and migrate to the bile duct where they mature; prolonged infection may result in cholangiocarcinoma [5]	Abdominal pain, watery stools. The patients in this case also had malaria; they also had malaria-like symptoms (fever, headache) [5]	Microscopy examination after staining the stools with Giemsa or Ziehl–Neelsen stains [5]	The authors encourage clinicians to consider clonorchiasis/opisthorchiasis infection a possible diagnosis for all undiagnosed abdominal pain since the infection has propensity to cause hepatic fibrosis, liver cancer and cholangiocarcinoma [5]
	Bleeding from the biliary tree is usually associated with trauma (can be iatrogenic), cholelithiasis, vascular disorders, tumors [39]	Hemobilia; tumor invasion into the intrahepatic bile duct, the tumor may rupture into the biliary system [39, 40]	Hematemesis and epigastric pain [39]	Abdominal CT [39] Multislice CT angiography is increasingly being used in the investigation [41]	Hemobilia may present as upper abdominal pain, gastrointestinal bleeding, and jaundice in some patients (22–38 %) [42]
Hepatic bile duct	Gallstones [6]	Biliary obstruction [6]	Pain in the RUQ +/– jaundice [6]	Abdominal ultrasound, ERCP [6]	
	Pigmented-stone formation and bacterial superinfection; *Ascaris lumbricoides* and Gram-negative bacteria consistent with *Klebsiella* species [43] "Through the portal circulation, larvae of *Ascaris lumbricoides *migrate from the stomach or small intestine to the liver and then to the lungs, where they break out into the airspaces, migrate up the trachea, and are swallowed. The larvae develop into sexually mature adults in the small intestine where they live and lay eggs that pass in the feces." [44]	Hepatolithiasis; inflammation in portal tracts, often with portal pylethrombophlebitis; patients with advanced disease have chronic lesions characterized by periductal fibrosis [43]	Recurrent pain in the RUQ of the abdomen [43]	Liver function test, abdominal ultrasound, CT, ERCP, MRI, MRCP, blood cultures, microscopy examination on stool specimen [43]	The 35-year-old patient was born in Vietnam and she immigrated to USA in her early 20s. Multiple large stones in the common bile duct, the left hepatic duct, and the left intrahepatic duct. A wide range of differential diagnoses were discussed in this article [43]. “Obstruction along the pancreatobiliary tree may occur if aberrant migration of larvae or adult worms through the ampulla of Vater occurs.” [45]
	Malignancy e.g. hepatocellular carcinoma [46]	Intrahepatic and extrahepatic bile duct obstruction [46]	Upper abdominal pain, jaundice and weight loss; icterus, and hepatomegaly [46]	Ultrasound and CT abdomen [46]	
	Malignancy in the intrahepatic biliary duct [47]	Intrahepatic cholangiocarcinoma [47]	Episodic epigastric pain and iron-deficiency anemia, jaundice [47]	Liver function test, full blood examination, iron studies, abdominal ultrasound, CT, and ERCP [47]	
	Intrahepatic perforated cholecystitis [11]	Intrahepatic abscess [11]	Intermittent fever, anorexia, and weight loss [11]	Ultrasound of the liver, and CT angiogram [11]	Other case [48]
	Cholesterol hepatolithiasis [49]	Heterotopic pancreatic tissues were distributed along the wall of the biliary tract and were composed of acinar cells and duct elements without islets of Langerhans [49]	Abdominal pain (45.5 %), epigastric discomfort (12.0 %), nausea and vomiting (9.6 %), bleeding (8.0 %) [49]	Ultrasound, CT scan, and definitive diagnosis by histopathological examination on the excised mass, ERCP [49]	The patient in the case was asymptomatic [49]
	Multifocal congenital dilatations of the intrahepatic bile ducts [50]	Caroli’s disease (the dilated intrahepatic ducts, which may be diffuse or limited, presenting in a sack form that produces cystic structures, which communicate with the biliary tree) [50]	Asymptomatic in this case [50]. Others have reported RUQ abdominal pain, jaundice, and recurrent cholangitis [51]	Abdominal CT, MRI, MRCP, histopathologic findings on resected liver specimen [50]	
	Obliterative cholangiopathy – biliary atresia [52]	Neonatal biliary obstruction and cholestasis [52]	Jaundice [52]	Urinary urobilinogen combined with GGT; liver biopsy; diagnosis of biliary atresia was confirmed by operative cholangiography and/or laparotomy findings [52]	
	Immune-mediated destruction of the intrahepatic bile ducts [53]	Primary biliary cirrhosis leads to decreased bile secretion and the retention of toxic substances within the liver, resulting in further hepatic damage, fibrosis, cirrhosis, and eventually, liver failure [53]	Fatigue and pruritus; unexplained discomfort in the RUQ of the abdomen; hepatomegaly; jaundice, portal hypertension and steatorrhea may occur in advanced disease; splenomegaly (uncommon); rarely ascites, hepatic encephalopathy, or hemorrhage from esophageal varices [53]	Antimitochondrial antibodies, which are present in 90–95 % of patients and are often detectable years before clinical signs appear; liver enzymes; liver biopsy [53]	
	Intense inflammatory fibrosis of the intrahepatic and extrahepatic biliary ducts [54]	Primary sclerosing cholangitis (PSC): fibrosis involving the common bile duct, hepatic ducts, and sometimes the gallbladder; may progress to secondary biliary cirrhosis [54]	Progressive obstructive jaundice, pruritus, weight loss, pain (RUQ or epigastric pain) [54]	Elevation of total and direct (conjugated) serum bilirubin, serum alkaline phosphatase were 3 to 5 times normal; operative cholangiograms [54]	PSC can be associated with inflammatory bowel disease. Primary biliary cirrhosis and sclerosing cholangiocarcinoma should be ruled out prior to diagnosing PSC [54]
	Extensive IgG4-positive plasma cells and T-lymphocyte infiltration of various organs including bile duct and gallbladder [55]	Systemic fibroinflammatory causing sclerosing cholangitis [55]	Painless jaundice and weight loss [55]	CT abdomen, liver function test, serum IgG4; “ERCP with intraductal ultrasonography (IDUS), brush cytology and endobiliary biopsy would be helpful” [55]	Another recent case reported that IgG4-related cholangitis is a rather uncommon cause of biliary obstruction, which can be easily mistaken for a cholangiocarcinoma [56]
	Abnormality in the genes encoding for canalicular bile formation [57]	Defective bile canaliculi leading to intrahepatic cholestasis which may progress to fibrosis and endstage liver disease (progressive familial intrahepatic cholestasis) [57]	Progressive jaundice [57]	Radiological, laboratory, and liver biopsy findings [57]	
	Cystic dilatation of extrahepatic bile ducts, with or without the dilatation of the intrahepatic duct [58]	Choledochal cyst [58]	In this case, an antenatal diagnosis of an abdominal cyst [58]. Patient can be asymptomatic [6]	Abdominal ultrasound and operative cholangiogram [58]. Liver function test, ERCP, CT or MRI [6]	Choledochal cyst may be associated with biliary atresia [59]
	Malignancy e.g. SCC at the bifurcation of the common hepatic duct, and adenocarcinoma in the common bile duct [60]	Biliary obstruction leading to hyperbilirubinemia [60]	Jaundice, dark urine, itch, and weight loss [60]	Abdominal ultrasound, CT, positron emission tomography-CT [60]	Other cases of malignancy at the common bile duct [61, 62]
Bile synthesis, conjugation, transport	Hepatic glucuronidating activity is reduced [63]	Mild, chronic unconjugated hyperbilirubinemia in the absence of hepatocellular disease or hemolysis (Gilbert syndrome) [63]	Intermittent mild jaundice [64]	Genetic testing [64]	Reduced expression of bilirubin UDP-glucuronosyltransferase 1 gene; an autosomal recessive mode of inheritance was suggested [63]
	Deficiency of hepatic glucuronyl transferase [65]	Hyperbilirubinemia of the unconjugated type; complication includes kernicterus (Crigler–Najjar syndrome) [65]	Jaundice since birth; hepatomegaly; frequent generalized, tonic and clonic convulsions due to kernicterus [65]	Serum bilirubin (direct/conjugated and indirect/unconjugated) test, genetic testing [65]	This disease is inherited as an autosomal recessive trait [65]
	A defect in the canalicular multispecific organic anion transporter (*cMOAT*) gene (*ABCC2/MRP2* superfamily) located at 10q24 [66]	Impaired hepatobiliary transport of non-bile salt organic anions leading to chronic conjugated hyperbilirubinemia (Dubin–Johnson syndrome) [66]	This patient presented with repeated episodes of jaundice during illness; other patients may present with abdominal pain, fatigue, liver enlargement, or dark urine [67] [68]	Liver function test, abdominal ultrasound, liver biopsy showed presence of parenchymal pigmentation, urinary coproporphyrin level, genetic testing [67]	This is an autosomal recessive disorder [67]
	Homozygous inactivation of two adjacent genes *SLCO1B1* and *SLCO1B3* encoding organic anion transporting polypeptides OATP1B1 and OATP1B3 [69]	Chronic conjugated hyperbilirubinemia without abnormal hepatic pigmentation (Rotor syndrome) [69, 70]. Abnormal transfer of sulfobromophthalein from plasma into the liver [70]	Nonhemolytic jaundice [71]	Urinary coproporphyrin and plasma sulfobromophthalein, genetic testing [70]	This is an autosomal recessive disorder [70]
Portal vein	Iatrogenic injury from surgical procedure [72]	Portal vein thrombosis [72]	Pain in the RUQ of the abdomen [72]	Abdominal CT and angiography [72]	Another case of portal vein thrombosis secondary to hyperhomocysteinemia with pernicious anemia was reported [73]
Hepatic artery	Iatrogenic injury from surgical procedure [72]	Hepatic ischemia [72], unrecognized vasculobiliary injury (VBI) can lead to biliary strictures, cholangitis and liver atrophy [74]	Pain in the RUQ of the abdomen [72]	Abdominal CT and angiography [72]	
Hepatic vein	Blood clots completely or partially block the hepatic veins that carry blood from the liver into the inferior vena cava [75]	Hepatic vein thrombosis/Budd–Chiari syndrome [75, 76]	Fatigue, abdominal pain, nausea, jaundice, hepatosplenomegaly, edema in the legs, ascites, and sometimes esophageal varices [75]	Doppler ultrasound examination of suprahepatic and cava veins [76]	Budd–Chiari syndrome is a vascular complication that can be associated with Behçet’s disease [76]
Sphincter of Oddi	Spasm or stenosis of the sphincter of Oddi (see note in the last column) [77]	Sphincter of Oddi dysfunction; idiopathic recurrent acute pancreatitis (can be controversial) [78]	Persistent or unexplained episodic abdominal pain in patients following cholecystectomy [77, 78]. The symptoms may precede cholecystectomy [77]	Sphincter of Oddi manometry [78]	Yaghoobi and Romagnuolo reviewed recent literature on sphincter of Oddi dysfunction; little is known about the etiology of the disease [78]
Ampulla of Vater	Adenoma at the ampulla [79]	Lithiasis of the bile duct and chronic pancreatitis [79]	Icterus/jaundice, and painless swelling of the gallbladder (Courvoisier sign) [79]	Abdominal ultrasound, CT, ERCP with biopsy of the lesion [79]	Adenoma has potential for malignancy [80]
Pancreas	Malignancy (e.g. tumor in the head of pancreas) [6]	The tumor compresses the common bile duct and pancreatic duct leading to hyperbilirubinemia [6]	Anorexia, weight loss, upper abdominal pain, and jaundice [6]	Abdominal CT and ERCP [6]	A recent case of a tumor in the head of pancreas [81]
Hematological process	Red blood cell enzyme (G6PD) deficiency [82]	The enzyme-deficient red blood cells are susceptible to hemolysis induced by certain drugs, bacterial and viral infections. Excessive hemolysis leads to increase in unconjugated bilirubin	Jaundice; generalized tonic–clonic seizure when kernicterus occurs [82]	Liver function test, serum G6PD level, G6PD deficiency phenotyping [82]	G6PD deficiency is an X-linked recessive disease
	Paroxysmal nocturnal hemoglobinuria	Prolonged and recurrent red blood cell breakdown results in increased bilirubin in the gallbladder. The excess bilirubin can precipitate bilirubin stones. Gallstones in the common bile duct [83]	Jaundice and abdominal pain [83]	CT, ERCP, MRCP [83]	
	Red blood cell membrane defect resulting in spherical, osmotically fragile erythrocytes (hereditary spherocytosis) [84]	Premature red blood cell destruction leading to hyperbilirubinemia [84]	Anemia, jaundice, and splenomegaly [84]	Peripheral blood smear showed small and dense spherocytes; osmotic fragility test [84]	The patient in the case has coexistence of hereditary spherocytosis and Gilbert syndrome [84]
Endocrinological process	Poorly controlled diabetes mellitus [85]	The excess glucose increases glycogen storage in the liver and blocks glycogenolysis resulting in glycogenic hepatopathy [85]	Hepatomegaly and pain in the RUQ [85]	Ultrasound abdomen, liver function test, and liver biopsy [85]	

This table is neither exhaustive nor comprehensive. For instance, it has not included drug-induced liver injury, and various infective causes such as hepatitis. It is meant to be a framework for discussion and illustration in this article. It can be deployed and continually updated by clinicians for their own use. ^a^In addition to a patient’s clinical history and physical examination findings. *CT* computed tomography, *ERCP* endoscopic retrograde cholangiopancreatography, *G6PD* glucose-6-phosphate dehydrogenase, *GGT* gamma-glutamyltransferase, *IDUS* intraductal ultrasonography, *IgG4* immunoglobulin G4, *MRCP* magnetic resonance cholangiopancreatography, *MRI* magnetic resonance imaging, *PSC* primary sclerosing cholangitis, *RUQ* right upper quadrant, *SCC* squamous cell carcinoma, *VBI* vasculobiliary injury

For example, the human biliary system is used for illustration (Table 1). Firstly, review the major anatomy and physiology of the biliary system, and list them in the first column. The anatomical figure of the biliary system (Fig. 2) will be helpful to provide visual cues to the physiological process. At each anatomical part or physiological process, we should explore what can be the potential failure or pathophysiological process, the corresponding effect(s), the resulting clinical features (symptoms and signs), investigation that can help a clinician to diagnose (in addition to the patient’s clinical history and physical examination), and special note. The treatment is not included in the table because the treatment option is dependent on the context of each individual. The note column is used for highlighting a key message or reminder, for example the authors of the case of clonorchiasis encourage clinicians to consider clonorchiasis or opisthorchiasis infection a possible diagnosis for all undiagnosed abdominal pain because the infection has the propensity to cause hepatic fibrosis, liver cancer and cholangiocarcinoma [5]. Also, the “note” column can be used to record citation of relevant new or additional cases (see Table 1).

**Fig. 2:**
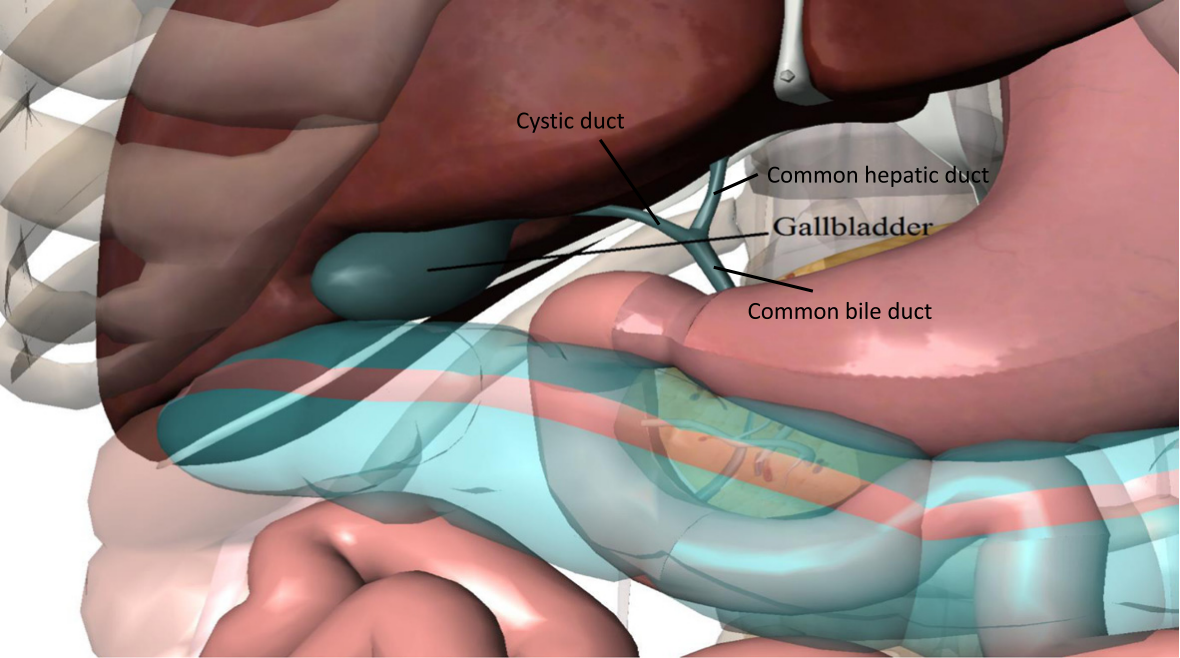
The human biliary system. Image courtesy of Visible Body (www.visiblebody.com)

Let us walk through the steps to establish a mFMEA in Table 1. You may refer to Table 1 and the references for citations of the relevant cases. Starting from the gallbladder, we have already known its common “potential failures” such as cholecystitis, cholecystolithiasis, and gallbladder cancer. By searching the medical literature, I have found some other failures such as gallbladder perforation, herniation, and torsion. The associated effects of the failures include obstruction, necrosis, and hernia. The main clinical features range from asymptomatic to abdominal pain with or without jaundice. The relevant investigations include abdominal ultrasound, computed tomography (CT) scan, endoscopic retrograde cholangiopancreatography (ERCP), and magnetic resonance cholangiopancreatography (MRCP).

In the cystic duct and common bile duct, obstruction (caused by gallstone or cancer) and infection are two common potential failures. In the hepatic duct (intrahepatic and extrahepatic duct), the potential failures include obstruction, infection, abscess, cancer, congenital-related abnormality, for example Caroli’s disease (congenital dilatation of the intrahepatic bile ducts), choledochal cyst, and biliary atresia, immune-mediated destruction of the intrahepatic bile ducts (primary biliary cirrhosis), intense inflammatory fibrosis of the intrahepatic and extrahepatic bile ducts (primary sclerosing cholangitis), immunoglobulin G4 (IgG4)-related cholangitis, and abnormality in the genes encoding for the bile canaliculi formation (progressive familial intrahepatic cholestasis). In addition to abdominal pain with or without jaundice, other symptoms and signs include systemic features such as fever, nausea, vomiting, anorexia, weight loss, anemia, fatigue, pruritus, steatorrhea, dark urine, and hepatosplenomegaly. Other investigations include liver function test, liver biopsy, antimitochondrial antibody, and serum IgG4 level according to the context of the patient.

In the process of bile synthesis, conjugation, and transport, the potential failures are broadly categorized into Gilbert syndrome, Crigler–Najjar syndrome, Dubin–Johnson syndrome, and Rotor syndrome. The clinical features range from asymptomatic to abdominal pain with or without jaundice. The investigations include special genetic tests, liver biopsy, and urinary coproporphyrin level, and plasma sulfobromophthalein depending on the clinical history of the patient.

Other anatomical parts that relate to the biliary system include portal vein, hepatic artery, hepatic vein, sphincter of Oddi, ampulla of Vater, and the pancreas. The potential failures include iatrogenic injury from surgical procedure, thrombosis, spasm, stenosis, and cancer. The clinical features include Courvoisier sign, legs edema, ascites, fatigue, anorexia, weight loss, abdominal pain, and jaundice. Special investigations include Doppler ultrasound of the suprahepatic and cava veins, abdominal CT and angiography, and sphincter of Oddi manometry.

The relevant potential failures of the hematological process, which is not restricted to a particular anatomical structure, include glucose-6-phosphate dehydrogenase (G6PD) deficiency, paroxysmal nocturnal hemoglobinuria (PNH), and hereditary spherocytosis. These abnormalities result in excessive hemolysis of red blood cells leading to hyperbilirubinemia. The clinical features include anemia, jaundice, splenomegaly, and kernicterus (in serious cases). The special investigations include serum G6PD level, genetic test, peripheral blood smear, and spherocyte osmotic fragility test.

An example of endocrinological condition has been reported in the New England Journal of Medicine - A patient with poorly controlled diabetes mellitus has excessive glucose in the blood leading to an increase in glycogen storage in the liver and inhibition of glycogenolysis resulting in glycogenic hepatopathy. The condition is manifested as hepatomegaly and pain in the RUQ of the abdomen.

After establishing the mFMEA in Table 1, I can update the relevant Ishikawa diagram for “pain in the RUQ of the abdomen” (Fig. 1) to include the detailed inputs for the “biliary pathology” as shown in Fig. 3.

**Fig. 3:**
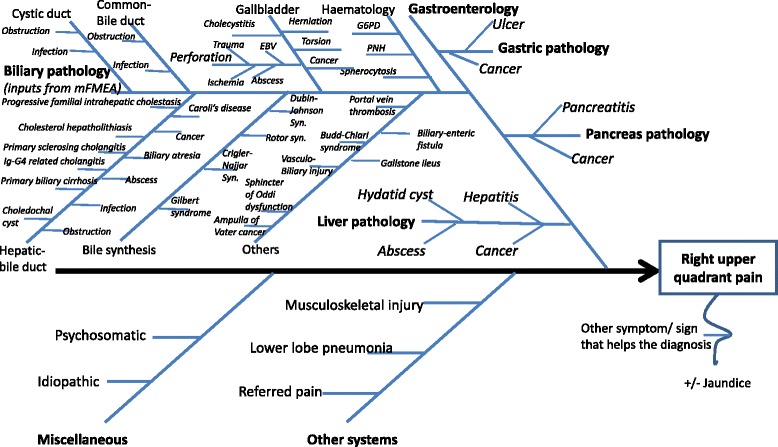
An Ishikawa diagram for a main presenting complaint of “abdominal pain in the right upper quadrant” with inputs from a modified failure mode and effects analysis. *mFMEA* modified failure mode and effects analysis

There are many sources of clinical cases such as:

- *Journal of Medical Case Reports*
- *BMJ Case Reports*
- *New England Journal of Medicine*
- Many other BioMed Central Open Access journals, for example *BMC Surgery*

How to keep a mFMEA table up to date.

- Set automatic notification when a relevant research or medical case report is published, for example setting a “search alert” in *Journal of Medical Case Reports*. This can be easily done by using the advanced search function, perform your search, and save the search history that you want to activate “search alert”. You can always go back to your “saved searches” to refine your search algorithm.
- Review an article and evaluate whether it fits into an anatomical or physiological part in a specific mFMEA, for example the biliary system. If a clinical case adds new findings in terms of pathophysiological process, unique clinical features, or investigation method, it should be added into the relevant columns in the mFMEA. This mFMEA process will collate and enrich the body of evidence for a specific clinical condition over time.
- You may attach a copy of the case report in the citation as a file attachment in an electronic database such as EndNote software. This will provide handy access to the references.
- The relevant main clinical features in the mFMEA, for example RUQ pain (or abdominal pain) can be extracted and reflected in the relevant Ishikawa diagram (see Figs. 1 and 3)

Advantages of weaving clinical cases into a mFMEA.

- mFMEA is a methodology that collates common, important, and critical (but rare) potential causes of a clinical condition.
- Studying clinical cases can reinforce clinicians’ reasoning and diagnostic skills, and clinical experience.
- Clinicians may not have the opportunity to be involved in caring for patients with various potential “failure modes” of a clinical condition. Studying clinical cases and weaving them into a mFMEA will provide the opportunity to substantiate the lack of experience.
- Medical educators can select relevant clinical cases from a mFMEA to set the scenario for teaching a relevant topic.
- Medical educators should encourage medical students to attempt the approach of identifying the potential pathophysiology and diagnosis before providing the answer. It is acceptable to err in role playing the clinical case, and learn from the errors!
- Interactive teaching and learning using clinical cases are more engaging and interesting compared to sole didactic teaching.
- Medical educators can relate the clinical cases to a relevant Ishikawa diagram and mFMEA.
- GPs/family physicians may use a mFMEA to identify and manage critical but rare conditions. They may not need to go into the details of certain pathophysiological processes which may not be relevant to their role as a GP/family physician, for example different types of gallbladder herniation. By contrast, surgeons could be interested to find out the various gallbladder herniations and surgical interventions reported in the literature to compare and enhance their clinical experiences.

## Concluding remarks

The mFMEA can be deployed as a tool to generate inputs for an Ishikawa diagram. Clinicians may apply the tool in their clinical reasoning process; while medical educators may select relevant clinical cases to set the scenarios to teach and facilitate a discussion among medical students, and relate the clinical cases back into a relevant mFMEA and Ishikawa diagram.
